# Proadrenomedullin N-Terminal 20 Peptide (PAMP) Increases Proliferation and Induces Cytoskeleton Remodeling in Melanoma Cells Through the CXCR7/CXCR4/β-Arrestin Axis

**DOI:** 10.3390/molecules31162791

**Published:** 2026-08-11

**Authors:** Tom Kalathil Raju, Pablo Garrido, Josune García-Sanmartín, Alfredo Martínez

**Affiliations:** Angiogenesis Group, Center for Biomedical Research of La Rioja (CIBIR), 26006 Logroño, Spain; tkalathil@riojasalud.es (T.K.R.); pgarridor@riojasalud.es (P.G.); jgarcias@riojasalud.es (J.G.-S.)

**Keywords:** PAMP, CXCR7, melanoma, tumor growth, migration, invasion, cytoskeleton remodeling, receptor inhibitors

## Abstract

Growth factors are molecules that interact with specific membrane receptors and induce cellular proliferation. In malignancy, this growth factor–receptor interaction constitutes one of the “hallmarks of cancer” and may result in uncontrolled cell growth. Proadrenomedullin N-terminal 20 peptide (PAMP) derives from proadrenomedullin and acts as an angiogenic peptide, and recent studies suggest it may be a tumor growth factor. In addition, it has been suggested that CXCR7 may be PAMP’s membrane receptor in particular cell types. Here, we combined advanced docking and molecular dynamic simulation studies to evaluate PAMP’s binding to CXCR7. Then, we used two melanoma cell lines and checked whether PAMP influences their shape, proliferation, migration, and invasion capacities. The signal transduction activated by PAMP was tested by Western blotting, and receptor involvement was investigated with specific inhibitors. This study shows that PAMP binds to active site 1 of CXCR7. It also increases melanoma cell proliferation through an autocrine growth loop. In contrast, PAMP reduced migration and invasion of these cells in a dose-dependent manner. PAMP was also responsible for modifying the actin cytoskeleton of both cell lines, producing a more elongated morphology. In addition, the presence of PAMP elevated ERK and AKT phosphorylation and increased CXCR7 and β-arrestin expression. Furthermore, specific inhibitors confirmed that these effects are mediated by the CXCR7/CXCR4/β-arrestin axis. In summary, we have shown that PAMP acts as a growth factor for melanoma cells while reducing their migration and invasion potential. These effects seem to be mediated by the CXCR7/CXCR4/β-arrestin axis. Altogether, these results suggest that PAMP inhibitors may be used as antitumor agents in melanoma.

## 1. Introduction

The characteristics that differentiate cancer cells from normal ones are known as the “hallmarks of cancer” [1]; among them, we find (i) sustained proliferative signaling, (ii) induced or accessed vasculature, and (iii) activation of invasion and metastasis [2]. The common link among these hallmarks is that, in general, they are initiated by growth factors (GFs), a group of peptidic molecules that are secreted to the extracellular milieu and bind specifically to high-affinity receptors on the cell surface [3]. The action of the GFs is very important for embryo development and the normal regulation of homeostasis [4] but, in the presence of oncogenes, they collaborate in inducing excessive cell growth, angiogenesis, invasion, and metastasis [5], thus contributing to cancer’s morbidity and mortality [6].

The relevance of GFs in cancer’s initiation and progression has resulted in the use of these peptides as disease biomarkers [7] and potential drug targets [8]. Nevertheless, even though there is a large number of drugs already available for cancer control, drug resistance is a growing concern, and the discovery and characterization of new GFs may open new avenues of pharmaceutical research.

Numerous families of GFs have been described. Those playing a significant role in tumor progression include the epidermal growth factor (EGF), platelet-derived growth factor (PDGF), vascular endothelial growth factor (VEGF), fibroblast growth factor (FGF) and transforming growth factor beta (TGFβ) families, among others [9]. Adrenomedullin (AM) and related peptides constitute another family of GFs. AM acts as a critical autocrine/paracrine molecule that promotes cardiac development [10], vasodilation, bronchodilation [11], regulation of hormone secretion [12], antimicrobial activities, growth regulation, and pro-angiogenesis [13]. The AM gene is located on the short arm of human chromosome 11 (or mouse chromosome 7), and the promoter region contains several protein-binding sites, including those for stimulator protein1, HIF1 and activator protein2 [14]. AM is generated through the proteolysis of a precursor molecule called proadrenomedullin, which also gives rise to another biologically active peptide, called proadrenomedullin N-terminal 20 peptide (PAMP) [15]. AM binds to a G-protein-coupled receptor (GPCR) named the calcitonin receptor-like receptor (CLR), which also requires interaction with one of three accessory membrane proteins called receptor activity modifying proteins (RAMPs 1–3) [16]. Although PAMP is derived from the same prohormone as AM, these peptides do not share either sequence similarity or binding receptors [17]. PAMP is a 20-amino acid peptide with a tight alpha helical shape [18]. Both peptides have an amidated carboxy terminus that is required for receptor binding and physiological actions.

A high expression of PAMP was found in the skin, adrenal medulla, heart ventricle, kidney, and lung, somehow mirroring that of AM [13]. PAMP is rapidly degraded by natural endopeptidases, and the carboxy-terminal amidation motif protects PAMP against proteolysis and prolongs its half-life [19].

PAMP was identified as a potent angiogenic factor that is active at concentrations of up to six orders of magnitude lower than other recognized proangiogenic peptides such as AM or VEGF [20]. However, there is limited research data on the potential impact of PAMP on tumor cell proliferation, migration, and invasion. An early report involving the neuroblastoma cell line TGW showed that both AM and PAMP reduced the proliferation potential of these cells [21]. Another study indicated that AM stimulated ^3^H-thymidine uptake in the teratocarcinoma cell line PA-1, whereas PAMP inhibited the AM-stimulated thymidine uptake in those cells [22]. Unfortunately, there are no studies available on PAMP in other more commonly studied cancer cell lines.

The receptors that transduce PAMP signaling have not been clearly characterized; however, two GPCRs, Mas-related G protein-coupled receptor member X2 (MrgX2) [23] and atypical chemokine receptor3 (ACKR3) [17], have been proposed as potential candidates. MrgX2 is predominantly found in neurons and several immune cells including mast cells, macrophages, dendritic cells, and T cells [24]. This receptor is relevant for immune regulation and is currently investigated as a potential target for cancer immunotherapy [25]. In contrast, ACKR3, also known as C-X-C chemokine receptor type 7 (CXCR7), is a non-classical GPCR which is present in several tumor cells and other cells of the tumor microenvironment. Furthermore, its expression levels are elevated in several cancers [26].

CXCR7 is a selective scavenger receptor for endogenous chemokines, CXCL11 and CXCL12, and the non-endogenous chemokine vCCL2 [26], as well as for the macrophage migration inhibitory factor (MIF) [27]. In addition, CXCR7 is a high-affinity scavenger for a broad spectrum of opioid and non-opioid peptides and modulates their availability for their respective receptors [17,26]. The expression of CXCR7 is ubiquitous but is especially abundant in various brain regions, adrenal glands, lymphatic and blood vasculature, heart, and various subsets of immune cells [26,27]. CXCR7 regulates embryogenesis, hematopoiesis, neuronal migration, angiogenesis, and cardiac development [27]. The endogenous chemokine CXCL12 binds to both CXCR7 and CXCR4; however, its affinity for CXCR4 is 10 times lower [28]. When CXCR4 is activated by a ligand, intracellular signaling could go either through classical G-proteins or through a β-arrestin (β-AR)-dependent paradigm [28]. In the case of CXCR7, since it lacks the conserved DRY-LAIV motif of G-protein activation system, signal transduction is limited to β-AR activation [26]. The behavior of CXCR7 is tissue- and context-dependent [27,29]. It plays an important role from the initial development and progression of cancer to the metastatic stage [29]. The CXCL12/CXCR7/CXCR4 axis has been involved in tumor development by favoring the adaptation, survival, and proliferation of cancer cells [30]. Moreover, the proangiogenic role of the CXCR7/CXCR4 axis, plus the ability of CXCL12 to up-regulate and synergize with VEGF, further supports their link with tumor development [30]. In consequence, targeting the CXCR7/CXCR4 axis may offer a promising new avenue for tumor management [31].

Beta-ARs are a family of multifunctional adaptor proteins that regulate the signaling cascades of some GPCRs, including CXCR7 and CXCR4 [32]. The agonist activation of CXCR7 promotes the recruitment of β-AR. Then, β-AR binds to CXCR4 and modulates its activity, resulting in receptor desensitization and potential endocytosis and recycling [33]. The receptor-bound β-ARs act as scaffolding proteins for the endocytic machinery as well as other signaling molecules [34]. Structural studies of β-ARs have demonstrated that they interact with the receptors either through finger-loop insertion and/or through an N-domain attachment [33]. The process results in the cleavage of β-AR’s C-terminal tail and promotes the conformational modification of the complex to facilitate further binding of specific partners. The key signaling pathways modulated by β-AR scaffolding include the mitogen-activated protein kinase (MAPK) family and the Src-dependent pathways [35]. The MAPK family, which consists of serine-threonine kinases, includes ERK, p38 kinases, and C-Jun N-terminal kinases, which regulate cell cycle progression, regulation of transcription, and apoptosis. The recruitment of C-Src, a member of the non-receptor tyrosine kinase family, promotes phosphorylation of ERK and AKT, which then prevent apoptosis while promoting mitogenic signals [34,35].

In a previous study, we demonstrated that a DNA vaccine against PAMP resulted in a significant reduction of tumor angiogenesis and the number of Ki67+ (proliferating) tumor cells in a melanoma mouse model [36], thus suggesting PAMP as a potential GF for this malignancy. In addition, CXCR7 mRNA expression is the highest among chemokine receptors in melanoma cell lines, and it directly correlates with malignancy potential [35].

Therefore, in this study, we aimed to characterize the effects of PAMP on melanoma cells, including growth, migration, invasion, and cytoskeleton remodeling. In addition, the mediation of the CXCR7/CXCR4/β-AR axis in these functions has been explored with specific inhibitors and Western blots.

## 2. Results

### 2.1. Molecular Docking Suggests That PAMP Has Better Binding Affinity for CXCR7 than for CXCR4

**Figure 1:**
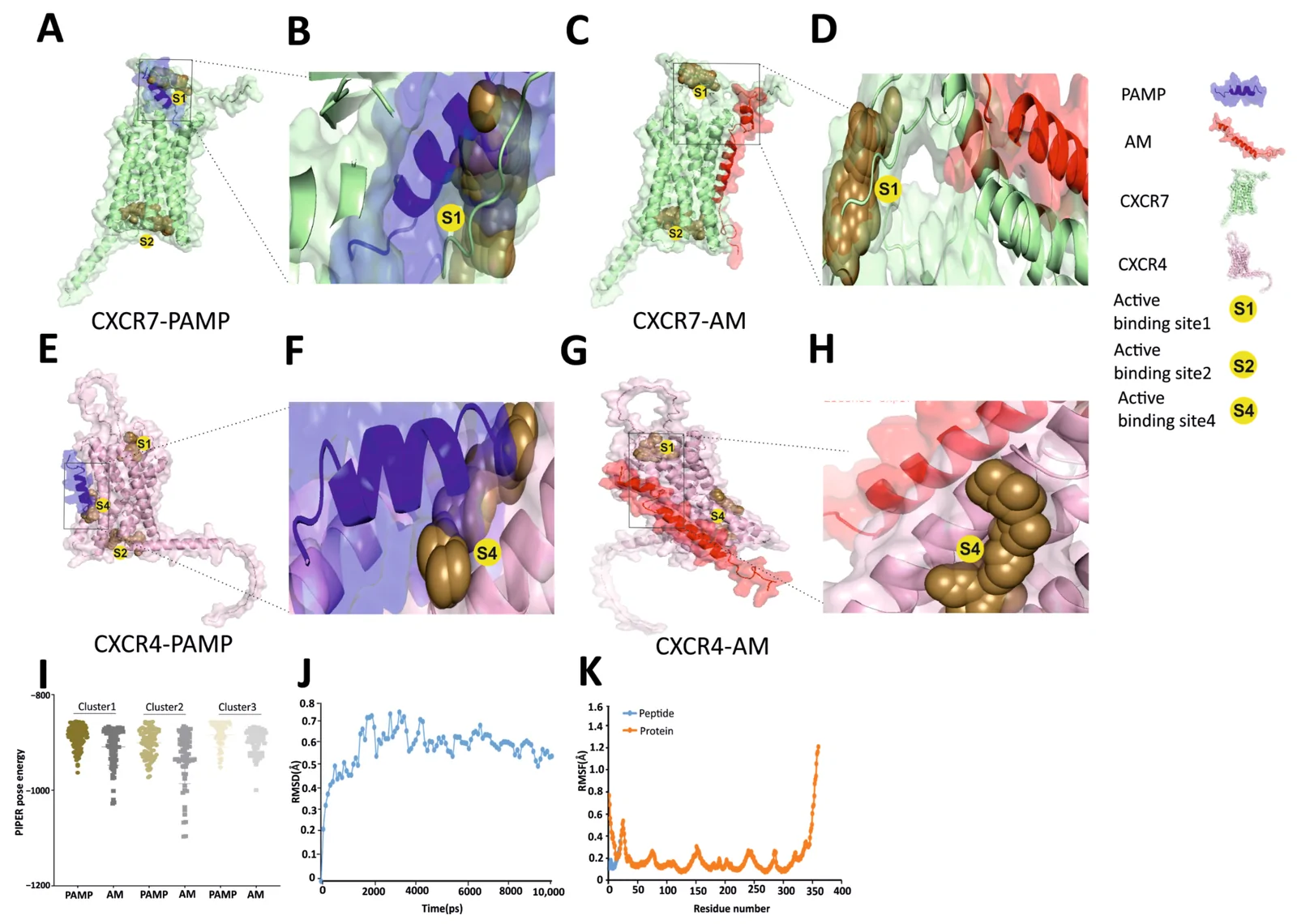
Representative docking images of PAMP (blue, (**A**,**B**,**E**,**F**)) and AM (red, (**C**,**D**,**G**,**H**)) interacting with either CXCR7 (green, (**A**–**D**)) or CXCR4 (mauve, (**E**–**H**)). The interactions between the ligands and the receptors were evaluated by the Mastero-Piper docking system. The combination yielding the highest cluster size for the optimal pose score for each pairing (155 for CXCR7/PAMP, 95 for CXCR7/AM, 170 for CXCR4/PAMP, and 84 for CXCR4/AM) was further examined with PYMOL and presented here. The active binding sites of the receptors are indicated with yellow circles. Docking pose energy plots of the top 3 clusters of PAMP and AM associated with CXCR7 are shown (**I**). Score cards for these representations can be found in Supplementary Material Tables S1–S4. Molecular dynamics: RMSD (**J**) and RMSF (**K**) plots of the PAMP–CXCR7 interaction demonstrate the high stability of the complex.

Protein structures for the peptides (PAMP, AM, and CXCL12) and the receptors (CXCR7 and CXCR4) were retrieved from Alphafold3, and molecular docking was performed with the aid of the Mastero program. The poses that obtained the lowest energies are represented in Figure 1A–H by using PYMOL software. The pose energies of the Top 10 clusters of the docking experiments are shown in Supplementary Tables S1–S4. The PAMP interaction with CXCR7 happens in the active binding site 1 (S1 site score = 1.046) (Figure 1A,B), which is the same place where the natural ligand, CXCL12, binds [31] (Supplementary Figure S1). In addition, the interaction of PAMP with CXCR4 happens in active binding site 4 (S4 site score = 0.854) (Figure 1E,F), while CXCL12 binds to both active binding sites 2 and 4 (Supplementary Figure S1). In contrast, the binding of AM does not coincide with the predicted binding sites of the receptors (Figure 1C,D,G,H).

To further characterize the binding of the peptides to the receptors, a pose energy plot of the top 3 clusters of the peptides with CXCR7 is shown (Figure 1I). All poses for PAMP were found around active site S1, whereas AM showed a more variable range of binding pose energies due to its non-fixed binding position (Figure 1I). The stable docked pose of PAMP with CXCR7 was further examined with Gromacs-based MD simulation analysis. A root mean square deviation (RMSD) plot for 10 ns showed that complex formation rapidly rises and attains a stable equilibrium at around 0.6 Å with minor fluctuations within the simulation time frame, denoting a structurally stable conformation (Figure 1J). Furthermore, the stable docked PAMP–CXCR7 complex was examined for its root mean square fluctuation (RMSF) backbone value plot, which showed a stable PAMP structure (blue, peptide) in the company of a CXCR7 protein (orange) with an overall stable N-terminal and core region and a highly flexible C-terminal loop (Figure 1K).

The score cards of the top 10 clusters for the different binding pairs are shown in Supplementary Material Tables S1–S4. Among the 1000 structures for each combination, the PAMP–CXCR7 pairs showed a better score weight than either AM–CXCR7 or PAMP–CXCR4, indicating that PAMP has a higher affinity than AM for this receptor and that PAMP binds preferentially to CXCR7 over CXCR4 (Supplementary Material Tables S1–S4).

### 2.2. PAMP Increases the Proliferation but Decreases the Migration and Invasion of Melanoma Cells

**Figure 2:**
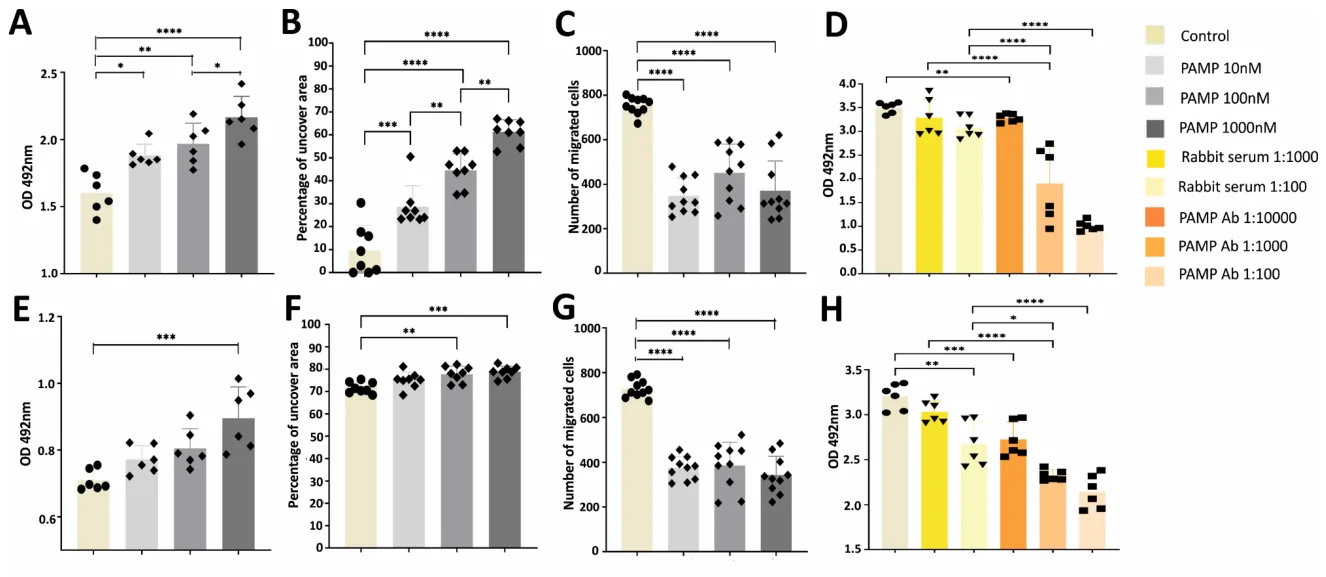
Analysis of PAMP effects on the proliferation, migration, and invasion of B16-F10 (**A**–**D**) and 5555 (**E**–**H**) melanoma cells. Proliferation (**A**,**E**), migration (**B**,**F**), and invasion (**C**,**G**) were quantified. The effects of the anti-PAMP antibody were tested (**D**,**H**), confirming the existence of an autocrine growth loop. Each bar represents the mean ± SEM of 6–10 observations. Statistical analysis: one-way ANOVA. *: *p* < 0.05; **: *p* < 0.01; ***: *p* < 0.001; ****: *p* < 0.0001.

To assess the impact of PAMP on the proliferation of melanoma cells, B16-F10 and 5555 cells were incubated with different concentrations of PAMP for different times. In B16-F10 cells, all concentrations tested resulted in significant increases in absorbance, showing a clear dose-dependent growth effect (Figure 2A) which was very significant at the highest PAMP concentration (*p* < 0.0001). The other cell line, 5555, showed a similar trend, although only the highest PAMP concentration presented a statistically significant growth (*p* < 0.001) (Figure 2E).

To investigate whether this growth effect may be part of an autocrine loop, we repeated these tests in the presence of a rabbit anti-PAMP antibody and a rabbit non-immune serum as a negative control. The anti-PAMP antibody was able to reduce tumor growth in both cell lines in a dose-dependent manner (Figure 2D,H). Although the higher dose (1:100) of the non-immune serum resulted in a slight reduction of tumor growth (*p* = 0.001) solely in 5555 cells, the 1:1000 dilution was undistinguishable from the untreated control. Furthermore, the 1:1000 dilution of the anti-PAMP antibody was significantly lower than the corresponding dilution of the non-immune serum (*p* < 0.0001) in both cell lines. This behavior clearly identifies the existence of an autocrine growth loop driven by PAMP in melanoma cells.

The influence of PAMP on the migration abilities of melanoma cells was further analyzed using a wound healing assay. The assay involved creating a scratch on a monolayer of cells and studying their movement into the “wound” over time. The wound gap for both melanoma cell types closed more slowly in the presence of PAMP when compared to controls (Figure 2B,F). The difference was more pronounced in B16-F10 than in 5555 cells, with the dose-dependent effect of PAMP beings more clear in the former (Figure 2B,F). Representative photographs of the migration assay in both cell lines are shown in Supplementary Figure S2A,B.

Furthermore, the effect of PAMP on the invasion capabilities of melanoma cells was evaluated using Matrigel-coated Boyden chambers. Although every concentration of PAMP notably diminished the invasion abilities in both cell lines (*p* < 0.0001), we did not see a dose-dependent response, probably because it was already maximized with the lower dose of PAMP (Figure 2C,G). Representative photographs of the invasion assay in both cell lines are shown in Supplementary Figure S2C.

### 2.3. PAMP Growth Effects Are Blocked by CXCR7 and CXCR4 Inhibitors

**Figure 3:**
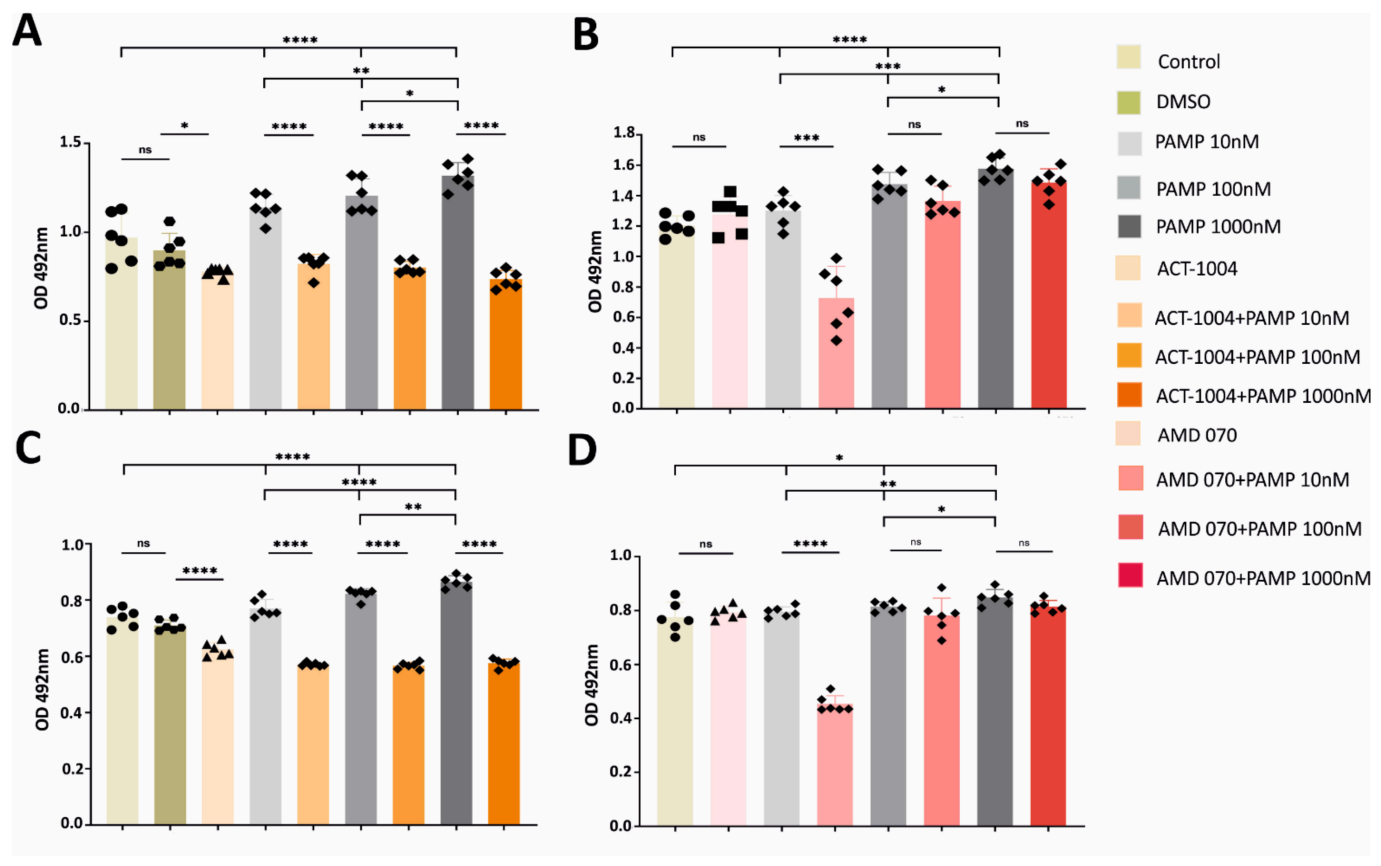
Analysis of PAMP effects on the proliferation of B16-F10 (**A**,**B**) and 5555 (**C**,**D**) melanoma cells in the presence or absence of 30 µM ACT-1004 (**A**,**C**) or AMD 070 (**B**,**D**) inhibitors. Each bar represents the mean ± SEM of 6 observations. Statistical analysis: one-way ANOVA. *: *p* < 0.05; **: *p* < 0.01; ***: *p* < 0.001; ****: *p* < 0.0001, ns = not significant.

To determine the effects of CXCR7 and CXCR4 inhibitors on the PAMP-induced growth changes on melanoma cells, we incubated those cells with increasing concentrations of PAMP in the presence or absence of the inhibitors. First of all, the incubation with the CXCR7 inhibitor ACT-1004, in the absence of PAMP, resulted in a significant growth reduction compared with the DMSO control in both cell lines, probably due to the existence of the PAMP-driven autocrine loop demonstrated above (Figure 3A,C). In contrast, this was not the case for the CXCR4 inhibitor AMD 070, which induced no changes compared to the control (Figure 3B,D). Furthermore, in the presence of PAMP, ACT-1004 completely blocked the PAMP-induced growth effects in both cell lines (*p* < 0.0001) (Figure 3A,C). This indicates the leading role of CXCR7 receptor in the function of PAMP on melanoma cells. On the other hand, the incubation with the CXCR4 inhibitor, AMD 070, resulted in a highly significant growth inhibition (*p* < 0.001) when used in combination with the lower concentration of PAMP (10 nM), whereas no changes were found with higher concentrations of PAMP. This happened in both cell lines (Figure 3B,D).

### 2.4. PAMP Modulates the Actin Cytoskeleton of Melanoma Cells

**Figure 4:**
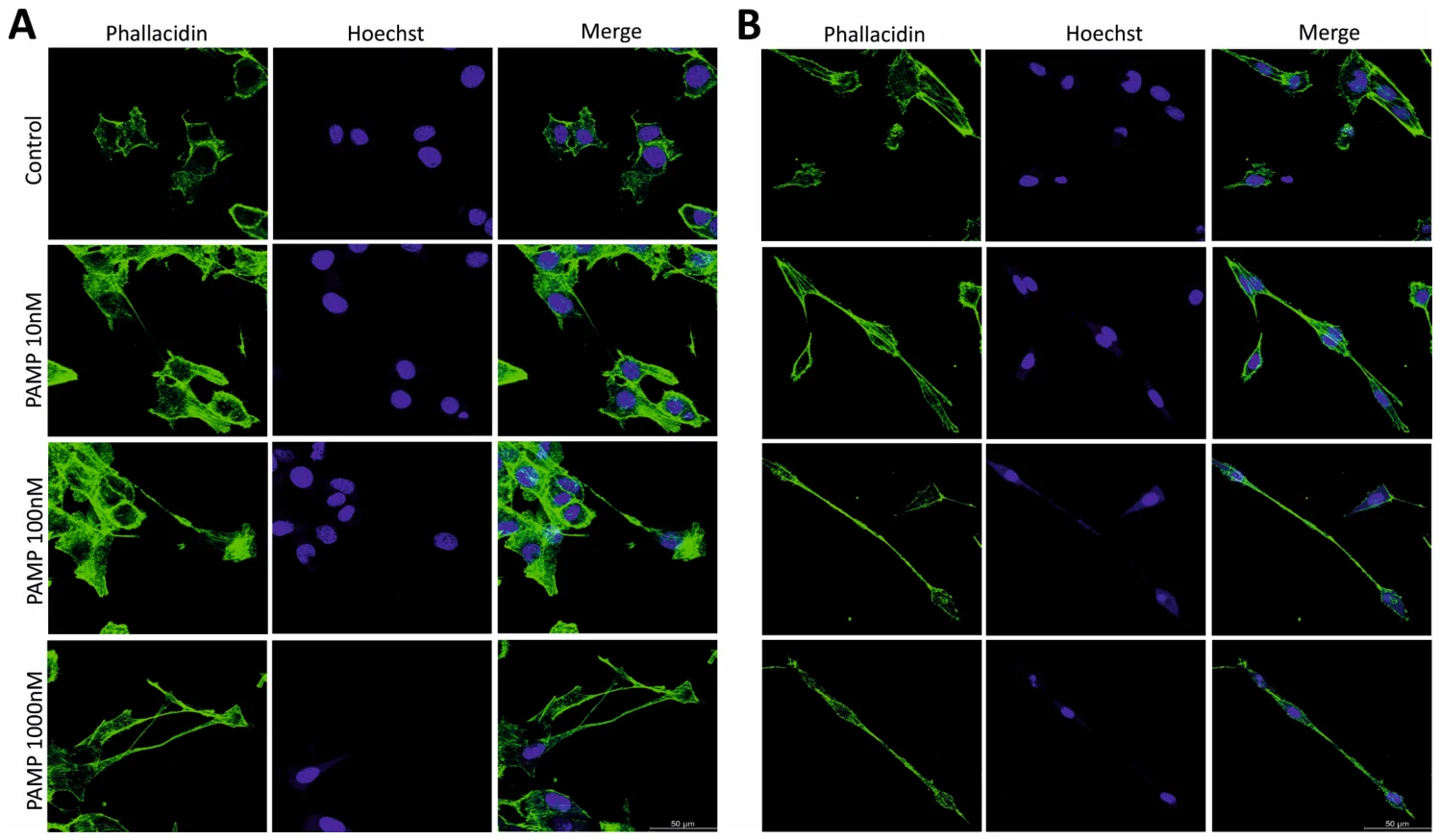
Effects of PAMP on the morphology of melanoma cells. Representative confocal images of melanoma cells B16-F10 (**A**) and 5555 (**B**) treated with different concentrations of PAMP. Green color shows actin cytoskeleton. Blue color shows the cell nuclei. Size bars = 50 µm.

While treating melanoma cells with PAMP in previous assays, we observed major changes in cell shape, favoring more elongated morphologies. To quantify these changes, cells were treated with increasing amounts of PAMP and, after 24 h of incubation, cells were stained with Bodipy-Phallacidin (which labels actin filaments in green) and Hoechst (which labels the nuclei in blue). Confocal microscopy images revealed that higher concentrations of PAMP led to an elongation effect compared to the untreated control cells in both cell lines (Figure 4).

**Figure 5:**
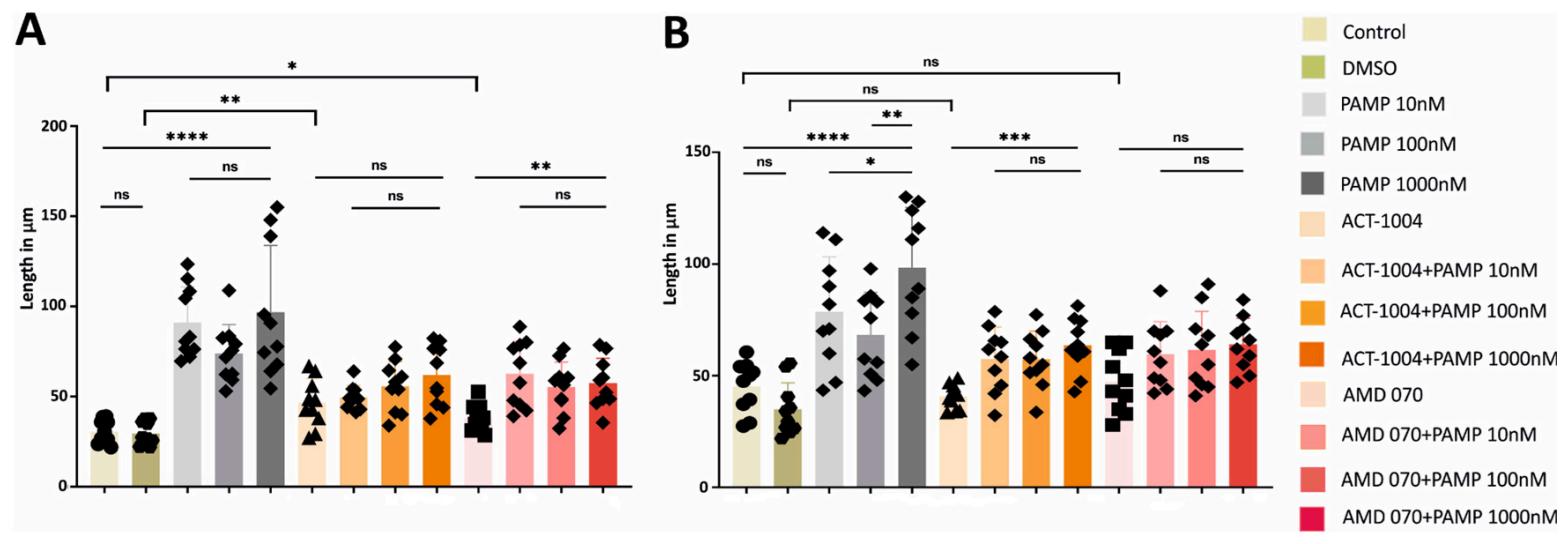
Quantitative analysis of morphological elongation due to PAMP on B16-F10 (**A**) and 5555 (**B**) melanoma cells in the presence or absence of 30 µM ACT-1004 and AMD 070 inhibitors. Each bar represents the mean ± SEM of 10 observations. Statistical analysis: one-way ANOVA. *: *p* < 0.05; **: *p* < 0.01; ***: *p* < 0.001; ****: *p* < 0.0001, ns = not significant.

To quantify the morphological elongation caused by PAMP on melanoma cells, we repeated the treatment with PAMP in the absence or presence of CXCR7 and CXCR4 inhibitors for 24 h, then stained the cells with crystal violet. Analysis of the microscopic images utilizing ImageJ indicated that the presence of PAMP resulted in significant elongation (*p* < 0.0001) in comparison to the untreated controls, in both cases (Figure 5A,B). In B16-F10, in the presence of 30 µM CXCR7 and CXCR4 inhibitors, the elongation was completely prevented with the CXCR7 inhibitor (*p* = 0.600), whereas some PAMP activity remained with the CXCR4 inhibitor (*p* = 0.003). In the case of 5555 cells, the reverse behaviors were observed, with the CXCR7 inhibitor being partially efficacious (*p* = 0.0007), whereas the CXCR4 inhibitor accomplished full blockade (*p* = 0.060). Representative pictures of crystal violet-stained cells are shown in Supplementary Figure S3. These results clearly implicate that both receptors have some impact in the elongation of the cell lines, although with some cell line specific nuances.

**Figure 6:**
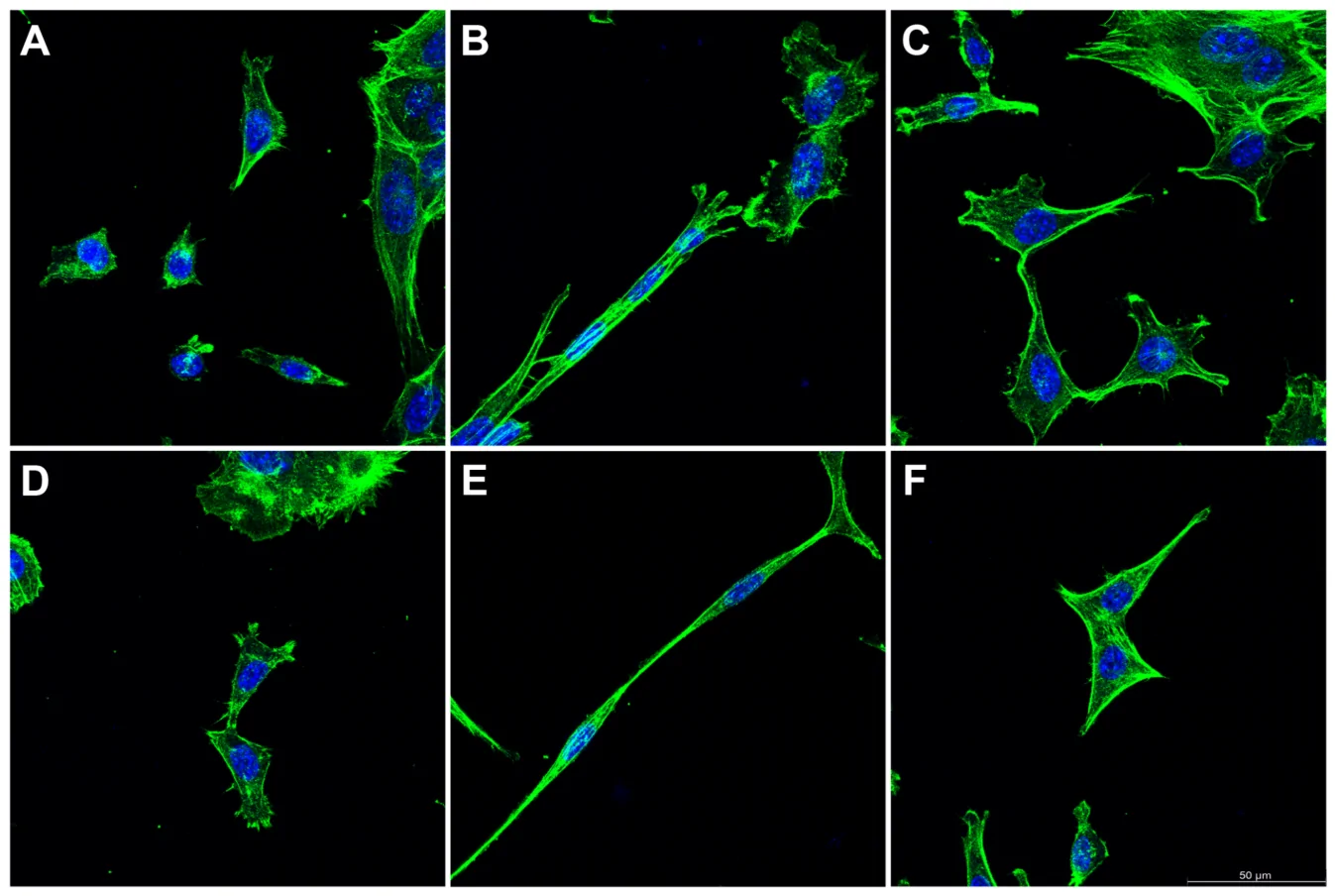
Representative confocal images of cell line 5555 exposed to untreated medium (**A**,**D**), 10 nM PAMP (**B**), 10 nM PAMP + AMD 070 (**C**), 100 nM PAMP (**E**), and 100 nM PAMP + ACT-1004 (**F**). Green color shows actin cytoskeleton. Blue color shows the cell nuclei. Size bar = 50 µm.

The action of the inhibitors on cell elongation was also confirmed with confocal microscopy (Figure 6). Both inhibitors reduced the length of the cellular prolongations (Figure 6C,F), although the cell morphology was not completely reverted to the one found in untreated cells (Figure 6A,B).

### 2.5. PAMP Increases ERK and AKT Phosphorylation Through the CXCR7/β-Arrestin Axis

**Figure 7:**
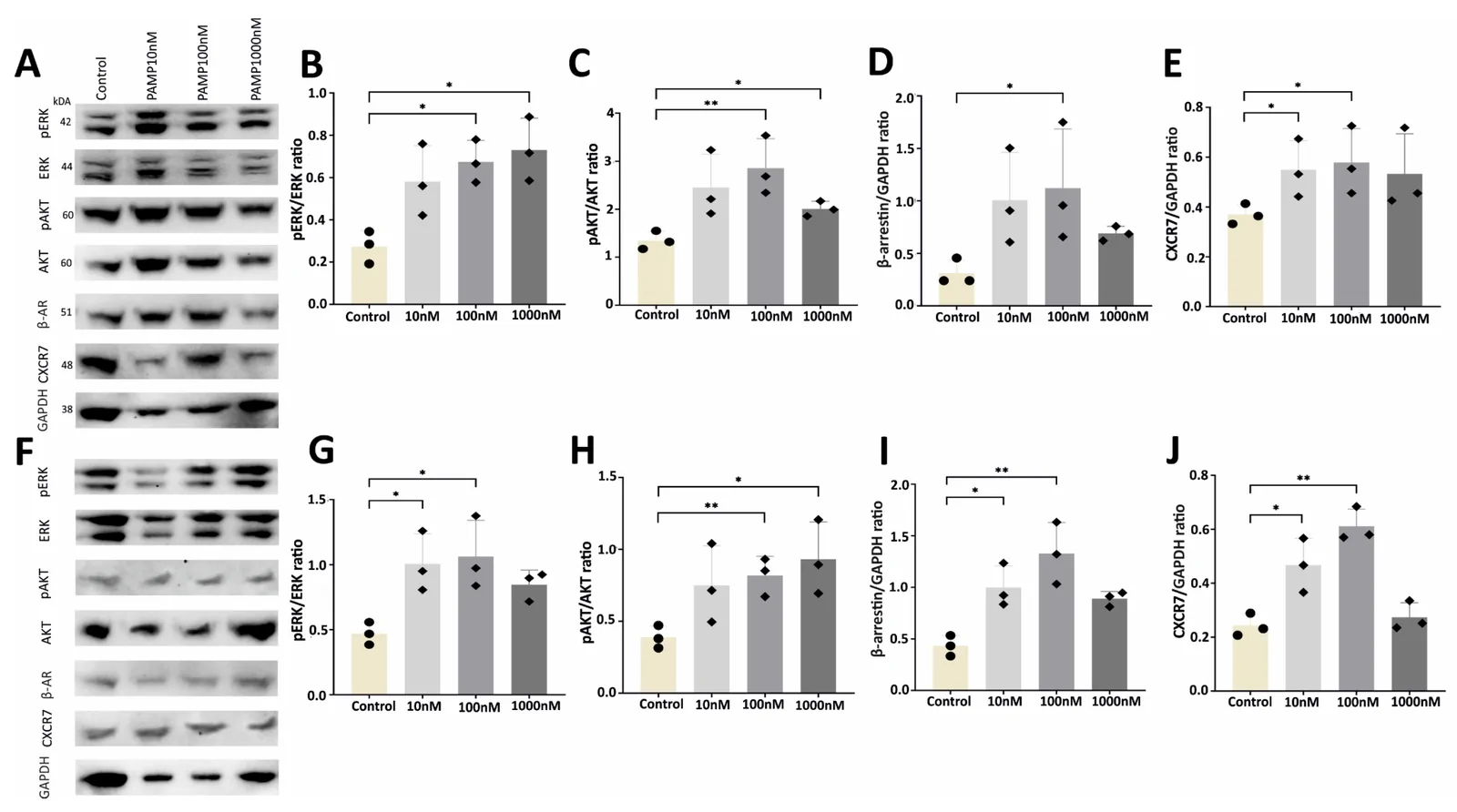
Molecular pathway analysis of the effects of PAMP on melanoma cells. Representative Western blots for B16-F10 (**A**–**E**) and 5555 (**F**–**J**) cell lines. Graphs display the normalized densitometry values for pERK/ERK (**B**,**G**), pAKT/AKT (**C**,**H**), β-AR/GAPDH (**D**,**I**), and CXCR7/GAPDH (**E**,**J**). Each bar represents the mean ± SEM of 3 observations. Statistical analysis: one-way ANOVA or Kruskal–Wallis. *: *p* < 0.05; **: *p* < 0.01.

The MAPK and AKT signaling pathways play a significant role in the growth and survival of cells, transmitting signals that originate from CXCR7 and CXCR4 receptors [35]. Consequently, we chose to investigate how PAMP affects the phosphorylation of ERK and AKT using Western blot analysis (Figure 7). Results from these experiments indicated that both kinases showed a marked increase in their phosphorylation levels when cells were treated with PAMP (Figure 7B,C,G,H). Additionally, PAMP enhanced the expression levels of β-AR when incubated with both cell lines (Figure 7D,I). Moreover, PAMP also elevated the expression of CXCR7 in both cell lines (Figure 7E,J). Interestingly, a dampened effect was observed in some cases at the highest PAMP concentration, suggesting a potential desensitization of the receptors. Western blots were also performed for CXCR4, but the expression of this protein was below the detection limit of the assay. Photographs of the whole blots are presented in Supplementary Figures S4–S7. These results indicate a contribution of these proteins in the signal transduction pathway initiated by PAMP and their responsibility for the physiological functions described above.

**Figure 8:**
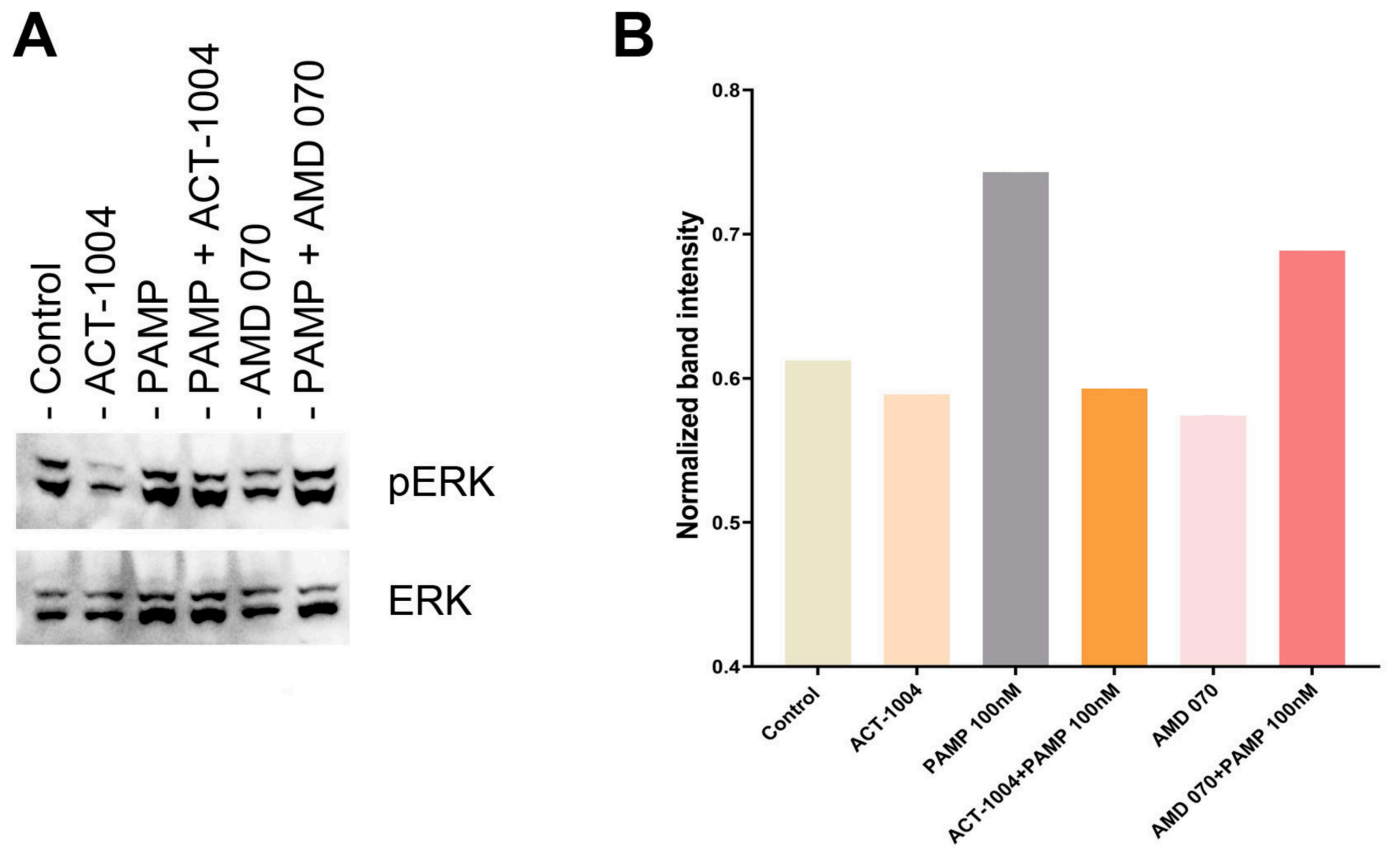
Preliminary Western blot analysis of cell line B16-F10 exposed to 100 nM PAMP in the presence/absence of 30 µM receptor inhibitors (**A**). Quantification of the ratio pERK/ERK of all bands (**B**). No statistical analysis was attempted.

Finally, to demonstrate that the kinase phosphorylation effect was also dependent on the CXCR7/CXCR4 receptors, we performed a preliminary experiment in which cell line B16-F10 was exposed to PAMP in the absence/presence of receptor inhibitors (Figure 8). Although we only ran a single blot and is impossible to establish statistical significance, results indicate that both inhibitors by themselves (in the absence of PAMP signaling) tend to reduce ERK phosphorylation, perhaps yet another indication of a PAMP-driven autocrine loop. Furthermore, the CXCR7 inhibitor ACT-1004 completely blocks PAMP-induced phosphorylation. In contrast, the CXCR4 inhibitor AMD 070 seems to reduce phosphorylation, but only partially, in agreement with the behavior we have seen in other experiments (Figure 8A,B). Photographs of the whole blots are presented in Supplementary Figure S8.

## 3. Discussion

In this study, we have demonstrated that PAMP binds to active binding site 1 of CXCR7 and, through this binding, it is able to significantly enhance melanoma cell tumor growth in a dose-dependent manner. This growth is driven by an autocrine loop, as demonstrated by the effects of PAMP antibodies on untreated cells. On the other hand, PAMP significantly delays tumor cell migration and invasion. Furthermore, the presence of PAMP induces an elongated morphology phenotype in the melanoma cells by modifying the actin cytoskeleton. All these functions were prevented by specific inhibitors of CXCR7 and CXCR4 receptors. Signal transduction studies showed that PAMP induced phosphorylation of ERK and AKT. All these functional attributes were confirmed in two mouse melanoma cell lines, B16-F10 and 5555.

The establishment and sustenance of neoplastic metastases are largely influenced by GFs [37]. The sequential stages in the early development of tumors are orchestrated by a variety of factors, including GFs [9]. Chemokines represent a specific category of GFs that play important roles in normal physiology and pathophysiology [37]. A key receptor system, known to significantly influence tumor proliferation and angiogenesis, is the CXCR7/CXCR4 axis [29]. The increased expression of CXCR7, and its associated partner CXCR4, has been linked to cell growth and tumor progression in various cancer types [38]. In this study, we have found that, in parallel with the natural ligand CXCL12, PAMP demonstrates a strong interaction with the CXCR7/CXCR4 axis and, in particular, with active site 1 of CXCR7. CXCR7 serves as an atypical chemokine receptor that functions as a scavenger for numerous chemokine and non-chemokine partners [26,28]. Earlier studies in kidney epithelial cells identified ACKR3 (= CXCR7) as a binding partner for AM, while other peptides derived from the proadrenomedullin precursor molecule, such as PAMP and the peptide fragment PAMP_(8–20)_, exhibited an even higher affinity for this scavenger receptor [17].

Our study has further validated the interaction of PAMP with the CXCR7 pathway in melanoma cells. Interestingly, our data suggest that the interaction between PAMP and CXCR7 does not appear to be in a scavenger capacity but instead seems to function as a classical ligand–receptor interaction, leading to the activation of signaling pathways and changes in cellular behavior.

The expression and secretion of AM has been shown across a wide range of tumor cell lines [13]. Some detailed studies with monoclonal antibodies confirmed that AM is an autocrine growth factor in malignant conditions since the same cells that express this peptide also have specific functional AM receptors [39]. Our growth experiments indicate that PAMP enhances the viability of melanoma cells in a dose-dependent fashion. The influence of dosage was more pronounced at 24 h than at later times, potentially reflecting the short half-life of short peptides [19,40]. The reduction in cell proliferation observed after incubation with PAMP antibodies, in the absence of externally added PAMP, indicates the existence of an autocrine loop similar to the one described for AM. This observation is also supported by a previous study where a DNA vaccine was produced against PAMP and tested in mice [36]. One of the main results of that study was the significant reduction of Ki67^+^ B16-F10 tumor cells in vaccinated animals, which already pointed to a proliferative effect of PAMP on these cells.

Our proliferation study in the presence of the CXCR7 inhibitor has shown that the PAMP-increased proliferation of melanoma cells was completely blocked in both cell lines, indicating the relevance of CXCR7 in PAMP pathophysiology. In the case of the CXCR4 inhibitor, the PAMP-induced proliferation was blocked only with the lowest concentration of PAMP (10 nM), but not with higher concentrations (100 and 1000 nM). This surprising result was very robust and happened in both cell lines. This observation may be explained by the biological response of the receptors to ligand availability. At low concentrations of the ligand, CXCR4 is fully functional and its inhibitor, AMD 070, works well and blocks biological responses. On the other hand, at higher concentrations, CXCR4 gets rapidly desensitized by internalization into the cell, while ligand-induced desensitization of CXCR7 is much lower, so CXCR7 takes over CXCR4 in an AMD 070-independent manner [31]. Obviously, since CXCR7 remains at the cell surface even at high ligand concentrations, its inhibitor, ACT-1004, was able to block PAMP’s biological activities at all concentrations tested.

In this study, the effect of PAMP on melanoma cells’ migration and invasion was also evaluated. There was a PAMP dose-dependent decrease in the migration of melanoma cells. This was more evident in B16-F10 compared to 5555 melanoma cells at 24 h. In addition, the incubation with PAMP made the melanoma cells less invasive. Thus, PAMP reduced invasiveness by 50% in both B16-F10 and 5555 melanoma cells after 24 h of incubation. Migration and invasion are two characteristics of tumor cells that are closely related to their metastatic potential [41]. Although in most cases, proliferation and migration/invasion go in the same direction, it is not uncommon to find biological examples where these two processes are inversely regulated. In fact, this phenomenon is known as the migration–proliferation dichotomy [41]. To explain this apparent discrepancy, the “go-or-grow” hypothesis has been proposed. Following this theory, some cells need to prioritize their energy expenditure towards either proliferation or migration/invasion but cannot perform both of them simultaneously with high efficiency [42]. This dichotomy seems to be driven by phenotypic switching, which could be determined by extracellular matrix cues [43], cell density, or by transcriptional elements such as c-Myc or NF-κB [44]. This behavior has been shown both in vitro and in vivo. In many cancers, hypoxic conditions and highly proliferative cells characterize the tumor center, whereas the periphery is exposed to stress factors that induce transition to a less proliferative, more migrating phenotype [45]. This phenomenon has implications for tumor treatment: since most chemotherapies rely on cytotoxic antiproliferative drugs, they may inadvertently exacerbate cell migration and invasion [46], suggesting that targeting both proliferation and migration simultaneously may be a more efficient antitumor strategy. Obviously, PAMP seems to push melanoma cells to a more proliferative behavior while sacrificing migration and invasion.

Tumor cells’ invasion and motility are directly connected to the dynamic remodeling of the cytoskeleton and extracellular matrix. Melanoma cells may undergo cell-shape changes, alternating between a rounder “amoeboid” shape and a more elongated “mesenchymal” morphology [47]. The amoeboid-to-mesenchymal transition is primarily induced by the NEDD9-DOCK3-Rac1 signaling pathway [48]. Different extracellular ligands have been shown to modulate this molecular cascade. For instance, TGF-β signaling promotes cell rounding, whereas TGF-β inhibitors result in more elongated cells [49]; meanwhile, WNT5A and integrin β3 induce the mesenchymal phenotype [50,51]. In addition, intracellular Ca^2+^ oscillations and mechanotransduction (YAP pathway) result also in melanoma cell elongation [52,53]. Interestingly, the NEDD9-DOCK3-Rac1 pathway is intricately connected to both AKT and ERK signaling networks, with ERK signaling driving NEDD9 expression and NEDD9 dynamically sustaining ERK activity [54]. In our study, we have demonstrated that PAMP induces AKT and ERK phosphorylation that, in turn, may activate NEDD9 and its downstream signaling cascade to induce the characteristic mesenchymal phenotype of melanoma cells.

In the same line of thought, melanoma cells can acquire an elongated morphology and even produce hollow tubes in 3D-culture conditions, a distinct feature known as vasculogenic mimicry [55]. This is very relevant since melanoma cells can substitute endothelial cells in the formation of new blood vessels, thus greatly facilitating tumor supply of oxygen and nutrients [56]. PAMP was characterized as a very efficient pro-angiogenic factor [20], and this may represent yet another side of the same function. When incubated with PAMP, melanoma cells developed more elongated shapes. This feature was present in both cell lines but was more prominent in 5555 cells. This effect was seen at different PAMP concentrations and was blocked by both CXCR7 and CXCR4 inhibitors. These findings further support the importance of the CXCR7/CXCR4 axis in melanoma cell differentiation and proliferation [26,38].

Our study also shows that incubating cells with PAMP significantly increases CXCR7 and β-AR expression, which may indicate the existence of a positive feedback loop. Furthermore, PAMP treatment increased ERK1/2 and AKT phosphorylation, which are well-known components of the signal transduction pathway of CXCR7/CXCR4 receptors [26]. Of course, phosphorylation of these molecules ultimately results in higher viability of melanoma cells, in agreement with our growth results.

That PAMP is a natural ligand of CXCR7/CXCR4 in melanoma cells was further supported by the up-regulation of β-AR. Rather than acting as a scavenger, PAMP seems to bind to CXCR7 as a *bona-fide* ligand, which then leads to the recruitment of β-AR. The recruited β-AR further activates the CXCR7 receptor and initiates signal transduction, thus creating a pool of activated melanoma cells rich in β-AR.

## 4. Materials and Methods

### 4.1. Molecular Docking and Simulation

The mouse sequence of all peptides and receptors was obtained from the Uniport database. Tertiary protein structures were predicted by using AlphaFold3 (https://golgi.sandbox.google.com, accessed on 10 February 2026), and the best model was selected from the five generated. The protein–peptide docking was performed using the PIPER module implemented in Maestro (Schrödinger suite windows version 2023-2, New York, NY, USA) [57]. The molecules were prepared for docking by using the protein preparation wizard with the OPLS4 force field. Hydrogen atoms were added, bond orders assigned, and structures minimized after optimization of hydrogen-bonding networks. Global rigid-body docking was carried out using the FFT-based PIPER algorithm, generating 70,000 poses that were clustered based on RMSD values. In all cases, the top 10 clusters based on their PIPER pose scores were selected. The best pose with more negative values (favorable binding) was selected from the generated models for visualization analysis using PyMOL (version 3.1.0).

The best docked protein–protein complex obtained from PIPER docking was subjected to molecular dynamics (MD) simulation using GROMACS (Windows version 2023.3) [58]. The system for simulation was prepared by using CHARMM-GUI webserver (https://charmm-gui.org, accessed on 13 March 2026) with the CHARMM 36 m force field and TIP3P water model [59]. The complex was then solvated in an orthorhombic periodic box and neutralized with 0.15 M NaCl. The energy minimization was performed using the steepest descent algorithm, followed by NVT and NPT equilibration at 300°K and 1 atm, respectively. A 10 ns production MD simulation was subsequently carried out with a 2 fs integration time step using the PME method for long-range electrostatics. Trajectory analyses including RMSD (backbone) and RMSF (backbone) were performed using GROMACS analysis utilities, and the results were plotted with Excel (Windows version 2013).

### 4.2. Cell Lines

Murine B16-F10 melanoma cells were acquired from the American Type Culture Collection (ATCC, Manassas, VA, USA), while the 5555 cells were derived from the BRAF-V600E mouse melanoma model [60] and were a generous gift from Dr. Imanol Arozarena (Navarra Biomed, Pamplona, Spain). Cells were maintained in RPMI 1640 (Lonza, Basel, Switzerland) supplemented with 5% fetal bovine serum (FBS, Gibco, Thermo Fisher Scientific, Waltham, MA, USA), and 1% penicillin/streptomycin (Gibco, Thermo Fisher Scientific, Waltham, MA, USA). The cultures were kept under a humidified atmosphere containing 5% CO_2_ at 37 °C and sub-cultured before they became confluent using a 0.25% trypsin/EDTA solution Lonza, Basel, Switzerland).

### 4.3. Peptide and Inhibitors

Synthetic mouse PAMP and a rabbit anti-mouse PAMP antibodies were custom-designed and made by Proteogenix (Schiltigheim, France) and have been used in previous studies [36]. The purity of the peptide reached 96.44%. An HPLC profile and mass spectrometry characterization of the peptide are shown in Supplementary Figure S8. The antibody was affinity-purified and characterized by ELISA against the antigen. The CXCR7 inhibitor ACT-1004-1239 (MedChemExpress, Monmouth Junction, NJ, USA) and the CXCR4 inhibitor AMD 070 hydrochloride (Sigma-Aldrich, St. Louis, MO, USA) were diluted in DMSO and water, respectively, as per manufacturer’s instructions.

### 4.4. Proliferation Assay

Melanoma cells were seeded in 96-well plates at a density of 2.0 × 10^3^ cells/well in RPMI medium containing 5% FBS and 1% antibiotic and incubated overnight. After that, cells were exposed to different concentrations of PAMP (10 nM, 100 nM, or 1000 nM) for incubation periods of 24, 48, and 72 h. To identify the effects of CXCR7 and CXCR4 inhibitors, cells were incubated with increasing concentrations of PAMP along with 30 µM of the respective inhibitor. Furthermore, cells were exposed to an anti-PAMP rabbit polyclonal antibody or control non-immune rabbit serum at different concentrations (1:10,000, 1:1000, and 1:100) for 24 h. At the end of the incubation period, the number of active cells was estimated with the colorimetric kit CellTiter96^®^ AQueous OneSolution (G3580, Promega, Madison, WI, USA), following manufacturer’s instructions. This solution contains a tetrazolium compound [3-(4,5-dimethyl-2-yl)-5-(3-carboxymethoxyphenyl)-2-(4-sulfophenyl)-2H-tetrazolium; MTS] and an electron coupling reagent (phenazine ethosulfate). In the presence of active mitochondria, the yellow tetrazolium salt is reduced into purple-colored formazan. The MTS solution was added to the plates (15 µL/well) that were then incubated at 37 °C and scanned at 492 nm every 30 min for 3 h with a POLARstar microplate reader (BMG Labtech, Ortenberg, Germany). For each condition, at least 6 replicates were analyzed.

### 4.5. Migration Assay

Melanoma cells were seeded in 6-well plates at 2.0 × 10^4^ cells/well one day prior to the experiment. Once the cells had reached 90–100% confluence, they were treated with serial concentrations of PAMP in medium containing 5% FBS, as before, and incubated for 1 h prior to wounding. At t = 0, each well was scratched with a 200 µL pipette tip, creating a “breaking line” design. Specific areas were labelled (4 areas/well) and photographed at 0, 24, 48, and 72 h after wounding using a DMI4000B inverted microscope (Leica Microsystems, Wetzlar, Germany) equipped with a DFC300 Fx digital camera (Leica Microsystems, Wetzlar, Germany). The surface that was not occupied by cells was subsequently analyzed and quantified using ImageJ v1.54 h (NIH, Bethesda, MD, USA). For each condition, at least 8 replicates were analyzed.

### 4.6. Invasion Assay

Sterile 24-Transwell plates containing cell culture inserts with 8-μm pores in a polycarbonate membrane (cat. Number 3422), and Matrigel matrix (cat. Number 354234), were procured from Corning (Corning, NY, USA). Always under sterile conditions, cell culture inserts were coated with 100 µL Matrigel, and plates were incubated at 37 °C for 2 h to ensure matrix solidification. The inserts were then loaded with 2.5 × 10^5^ cells in a volume of 500 µL of RPMI media with 5% FBS, and the lower chamber was filled with 750 µL of media with or without different concentrations of PAMP (10 nM, 100 nM, or 1000 nM) and incubated overnight at 37 °C. The next day, the non-invading cells were removed from the Matrigel-coated insert with a moistened cotton swap. Then, cells were fixed for 2 min in 100% methanol and stained with 1% crystal violet solution. To quantify invasion, 100× images of the inserts were taken with a DMI6000B microscope (Leica Microsystems, Wetzlar, Germany) and were further analyzed with the ImageJ v1.54 h program. For each condition, at least 2 replicates were analyzed. The process involves converting images to 8-bit, applying a threshold to select stained areas, and counting and recording the invading cells.

### 4.7. Crystal Violet Staining

Melanoma cells were seeded on sterile glass coverslips in 24-well plates at a density of 1.0 × 10^4^ cells/well, incubated until they were 50–70% confluent, and further exposed to serial concentrations of PAMP (10 nM, 100 nM, or 1000 nM) for 24 h. The next day, after thorough PBS rinses, cells were fixed with 10% buffered formalin for 10 min and permeabilized with 0.1% Triton X-100 in PBS for another 10 min. Following three washes with PBS, cells were stained with 1% crystal violet solution and mounted on glass slides. To identify the effects of CXCR7 and CXCR4 inhibitors, cells were incubated with increasing concentrations of PAMP along with 30 µM of the respective inhibitor. For each slide, 10 random images were taken with a DMI6000B microscope under 200× magnification, and further analyzed with the ImageJ v1.54 h program. The process involves calibration of the scale bar to microns and calculating the cellular length of 5–10 cells per image. For each condition, at least 2 replicates were analyzed.

### 4.8. Immunofluorescence

Melanoma cells were plated at 2.0 × 10^4^ cells/well in sterile glass-bottom microwell dishes (P35GC-1.5-14-C, Matteck Corportation, Ashland, MA, USA) in RPMI medium with 5% FBS and allowed to attach overnight. The next day, cells were exposed to serial concentrations of PAMP (10 nM, 100 nM, or 1000 nM), in the presence or absence of receptor inhibitors at 30 µM, and incubated for 24 h. The following day, after thorough PBS rinses, cells were fixed with 10% buffered formalin for 10 min and blocked with 0.1 M glycine solution for 30 min. Then, cells were stained with Bodipy-Phallacidin (41B1-2, Molecular Probes, Invitrogen, Waltham, MA, USA) 1:500 and Hoechst 3342 (B2261, Sigma-Aldrich) 1:1000 in PBS for 30 min. Plates were observed and representative images were taken in a Leica TCS SP5 confocal microscope (Leica Microsystems, Wetzlar, Germany).

### 4.9. Western Blot Analysis

Melanoma cells (5.0 × 10^4^ per well) were plated on 24-well plates overnight. The next day, cells were exposed to serial concentrations of PAMP (10 nM, 100 nM, or 1000 nM), in the presence or absence of receptor inhibitors at 30 µM, for 24 h. Each condition was tested in triplicates. The following day, cells were dispersed with 100 µL mammalian cell lysis buffer (Invitrogen, Waltham, MA, USA) supplemented with Complete Ultra tab (5892791001) and PhosSTOP tab (4906837001) as per the manufacturer’s instructions (Roche Diagnostics, Basel, Switzerland). Protein content was quantified using the bicinchoninic acid assay kit (Invitrogen, Waltham, MA, USA). A fixed amount of total protein (12.5 µg) was then mixed with appropriate amounts of 10× Nupage sample reducing buffer, 4× loading buffer (Invitrogen, Waltham, MA, USA) and water, as needed. The mixture was heated at 70 °C for 10 min, and separated by SDS polyacrylamide gel electrophoresis in 4–12% Bis-Tris gels (Invitrogen, Waltham, MA, USA). Prestained Seeblue plus protein ladder (LC5925, Invitrogen, Waltham, MA, USA) was used to estimate proteins’ molecular weights. Electrophoresed proteins were transferred to PVDF membranes using the iBlot 2 Transfer Device (Invitrogen, Waltham, MA, USA). Once transferred, the membranes were blocked with either 5% (*w*/*v*) milk or bovine serum albumin (BSA) in Tris-buffered saline (TBS, Invitrogen, Waltham, MA, USA) for 1 h and further exposed to either primary rabbit or mouse poly/monoclonal antibodies against pERK (4370, Cell signaling, Danvers, MA, USA), ERK1/2 (4695, Cell signaling), pAKT (9018, Cell signaling), AKT-pan (4691, Cell signaling), CXCR7 (CSB-PA001850, Cusabio, Houston, TX, USA), β-AR (CSB-RA568083A0HU, Cusabio, Houston, TX, USA), or GAPDH (SC-32233, Santa Cruz, Dallas, TX, USA) at a 1:1000 dilution overnight at 4 °C. On the next day, after three washes with TBST (1× TBS with 0.01% (*v*/*v*) Tween-20) and a final one in TBS, blots were incubated with horseradish peroxidase (HRP)-linked secondary antibodies to rabbit (7074, Cell Signaling, Danvers, MA, USA) or mouse (715-035-151, Jackson Immunoresearch, West Grove, PA, USA) IgGs at a 1:5000 dilution for 1 h. Peroxidase activity was detected with ECL Prime Western Blotting Detection Reagent (MB40201, NZYtech, Lisbon, Portugal) and captured in a ChemiDoc MP Imaging System (BioRad, Hercules, CA, USA). The quantification of the immunoreactive bands was accomplished with ImageLab software Version 6.0.1 (BioRad, Hercules, CA, USA).

### 4.10. Statistical Analysis

The datasets that possessed a normal distribution were analyzed by one-way ANOVA, and the detailed differences were identified by the post hoc Tukey test. For the datasets that did not follow a normal distribution, the Kruskal–Wallis test was applied and the specific differences were determined through Dunn’s post hoc test. All the analyses were performed utilizing GraphPad Prism v8.0 for Windows (GraphPad Software, San Diego, CA, USA). The data are presented as mean ± SEM and a *p* value < 0.05 was considered statistically significant.

## 5. Conclusions

In conclusion, we have shown that PAMP plays important roles in the pathophysiology of melanoma cells by increasing their growth, reducing their migration and invasion capacities, and modifying their actin cytoskeleton. All these activities are mediated by the CXCR7/CXCR4 receptors and the phosphorylation of ERK and AKT proteins. This peptide, which is also an important angiogenic factor [20], thus represents an interesting therapeutic target for melanomas and other malignancies. Small molecules [61], antibodies [62], peptide fragments [20], and a DNA vaccine [36] have been proposed as potential pharmacological approaches to reduce PAMP actions in cancer.

## Data Availability

The original contributions presented in this study are included in the article/Supplementary Materials. Further inquiries can be directed to the corresponding author.

## Supplementary Materials

The following supporting information can be downloaded at: https://www.mdpi.com/article/10.3390/molecules31162791/s1, Docking of CXCL12 to CXCR7 and CXCR4; Photographs of migration and invasion analyses; Representative crystal violet photographs; Full Western blots; HPLC and mass spectrometry analyses of synthetic PAMP peptide; and score cards for ligand/receptor docking.

## Abbreviations

The following abbreviations are used in this manuscript:

ACKR3	Atypical chemokine receptor 3
AKT	Protein kinase B
AM	Adrenomedullin
AP2	Clathrin adaptor protein
β-AR	Beta arrestin
CLR	Calcitonin receptor-like receptor
CXCR4	C-X-C chemokine receptor type 4
CXCR7	C-X-C chemokine receptor type 7
EGF	Epidermal growth factor
ERK	Extracellular signal-regulated kinase
FBS	Fetal bovine serum
FGF	Fibroblast growth factor
GF	Growth factor
GPCR	G protein-coupled receptor
HIF	Hypoxia inducible factor
MAPK	Mitogen-activated protein kinase
MD	Molecular dynamics
MIF	Migration inhibitory factor
MrgX2	Mas-related G protein-coupled receptor member X2
PAMP	Proadrenomedullin N-terminal 20 peptide
PDGF	Platelet-derived growth factor
RAMP	Receptor activity modulating protein
RMSD	Root mean square deviation
RMSF	Root mean square fluctuation
TFβ	Transforming growth factor beta
VEGF	Vascular endothelial growth factor
