# Supplementary material for: Proadrenomedullin N-Terminal 20 Peptide (PAMP) Increases Proliferation and Induces Cytoskeleton Remodeling in Melanoma Cells Through the CXCR7/CXCR4/β-Arrestin Axis
Source: Molecules. 2026 Aug 11;31(16):2791. doi: 10.3390/molecules31162791 (PMC13515915; doi:10.3390/molecules31162791)
Supplement: Supplementary file 1 [file molecules-31-02791-s001.zip › molecules-4390961-supplementary.pdf]

Supplementary materials

Proadrenomedullin N-terminal 20 peptide (PAMP) increases proliferation and induces cytoskeleton remodeling in melanoma cells through the CXCR7/CXCR4/β-arrestin axis

Tom Kalathil Raju, Pablo Garrido, Josune García-Sanmartín, Alfredo Martínez*

Supplementary figures

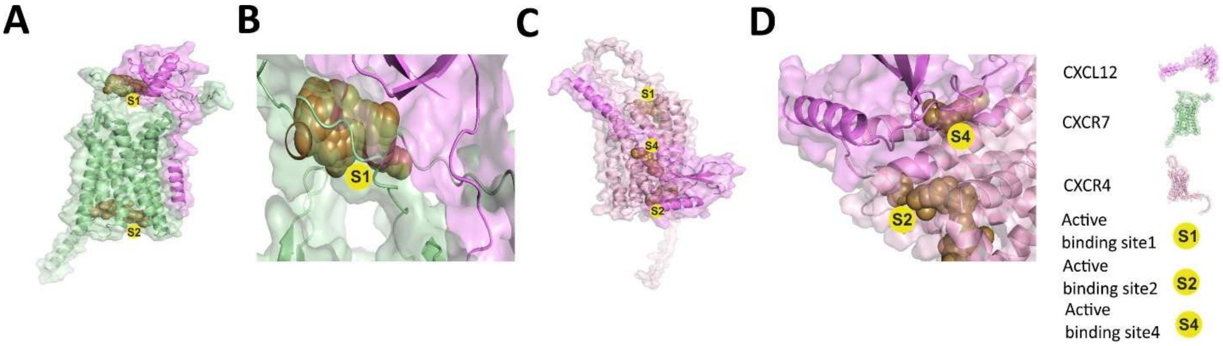

Suppl. Figure S1: Representative images of the CXCL12 docked poses with CXCR7 (A) with its magnified version (B) shows its interaction towards predicated dominant binding sites S1 (site score: 1.046). The interaction of the CXCL12 with CXCR4 (C) with its magnified version (D) shows its interaction towards predicted non-dominant binding sites S2 and S4 (site scores: 1.016 and 0.854, respectively).

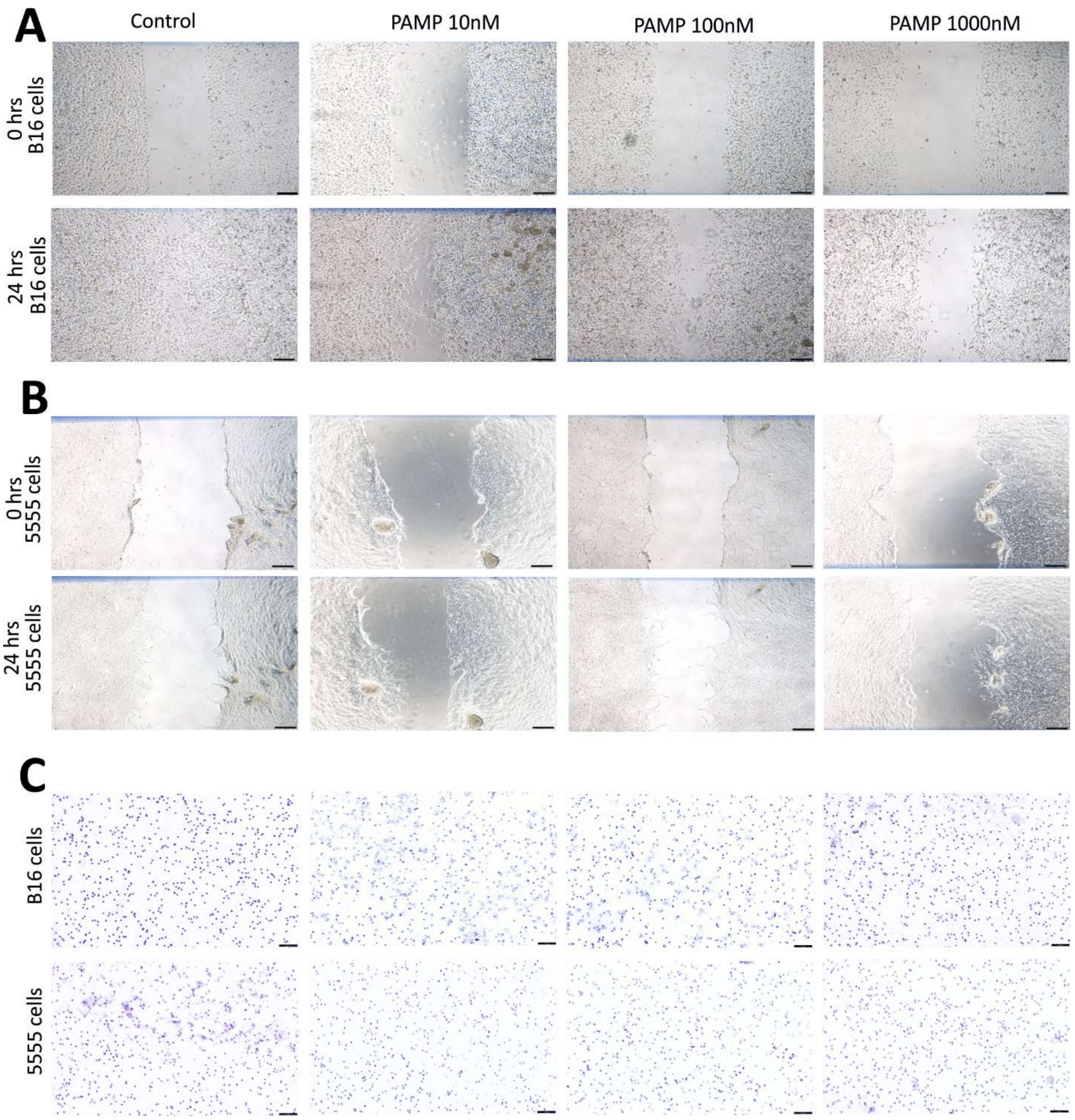

Suppl. Figure S2: Representative photographs of the migration (A,B) and invasion (C) assays. In the migration assays, cell lines B16-F10 (A) and 5555 (B) are represented at 0 and 24 h after inflicting the bound, with the different PAMP treatments. The pictures were taken at 400x magnification and analyzed by ImageJ program. Scale bars = 25 µm.

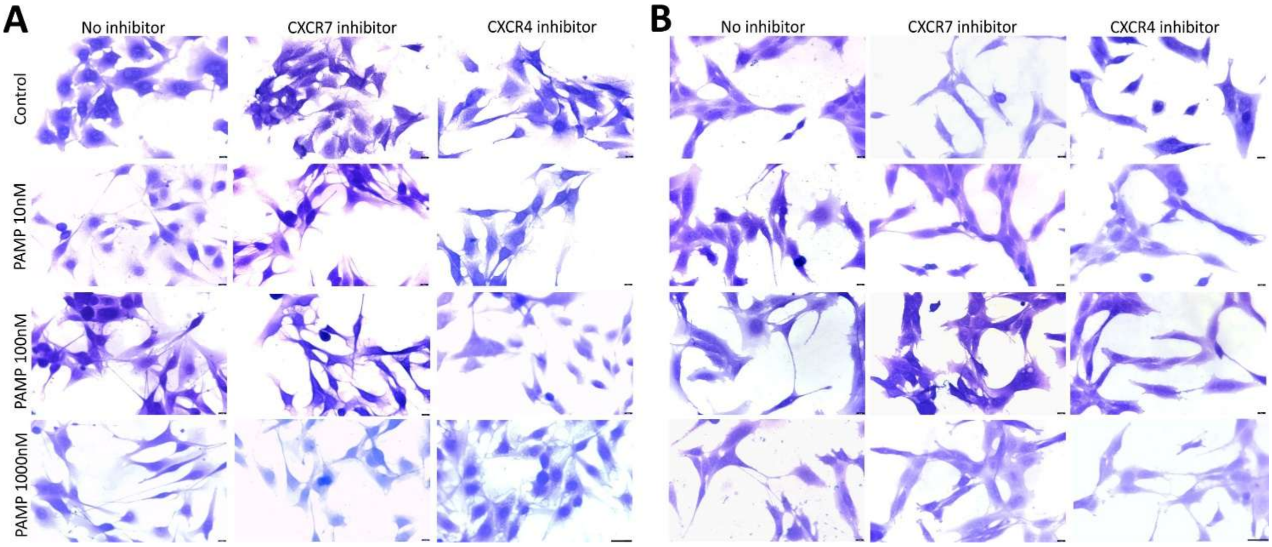

Suppl. Figure S3: Representative pictures of the crystal violet stained slides of the both B16-F10 (A) and 5555 (B) melanoma cell lines treated with increasing concentrations of PAMP with or without the presence of 30µM CXCR7 and CXCR4 inhibitors. The pictures were taken at 400x magnification and analyzed by imageJ program. Scale bars = 25 µm.

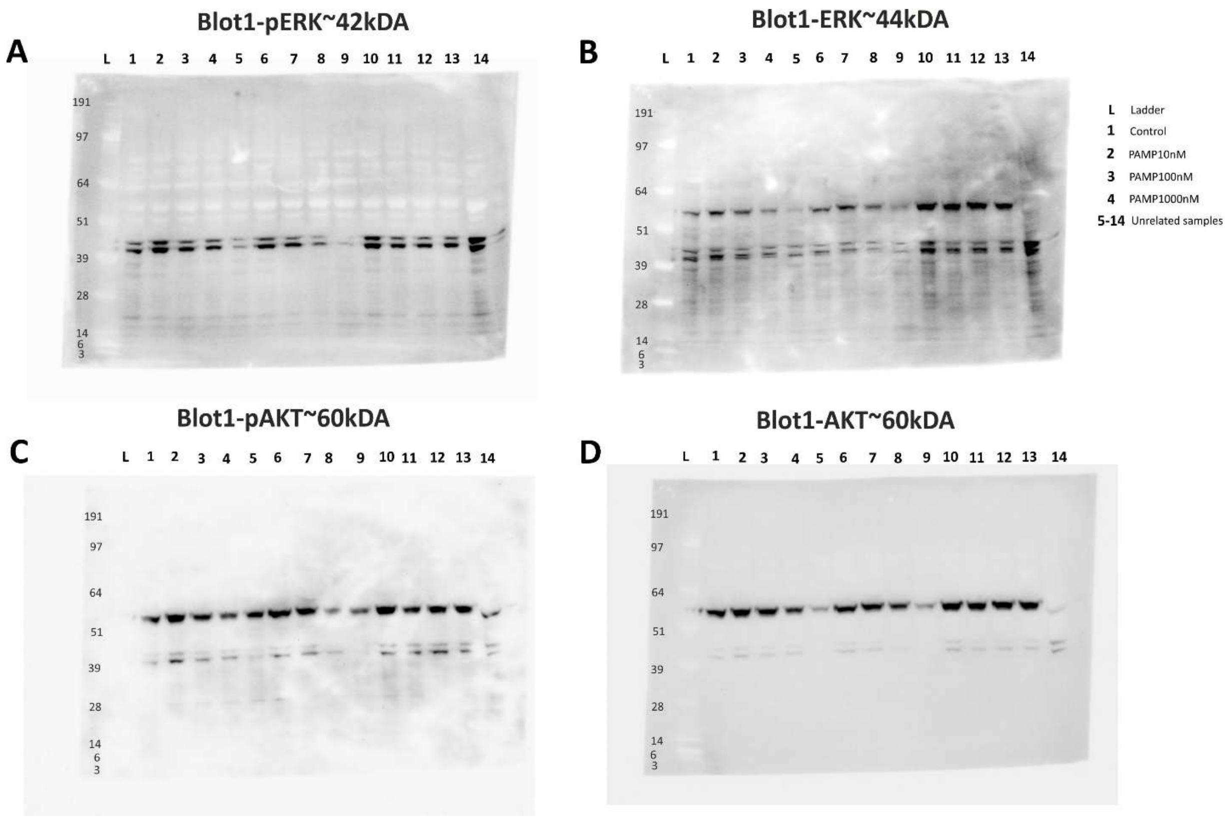

Suppl. Figure S4: Representative Western blots (full) of B16-F10 cell lysate analysis (A-D) after 24hrs of incubation with increasing concentrations of PAMP, as indicated. The blots were further incubated with the respective antibodies to detect the specific bands and images were taken by ChemiDoc MP Imaging System (Biorad). Prestained see blue plus ladder was used to estimate proteins´ molecular weight and visualized under normal white light (see Fig. S4D).

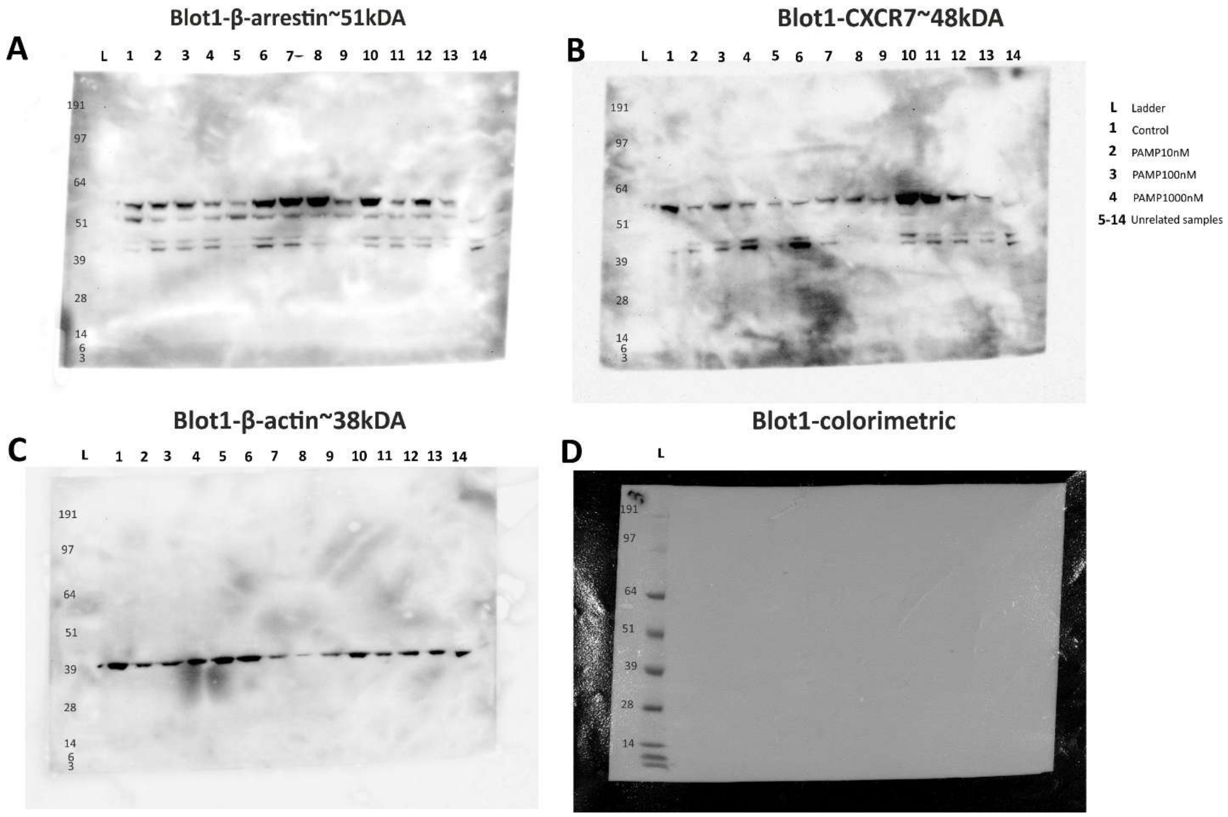

Suppl. Figure S5: Representative Western blot (full) of B16-F10 cell lysate analysis (A, B and C) after 24hrs of incubation with increasing concentrations of PAMP, as indicated. The blots were further incubated with the respective antibodies to detect the specific bands and images were taken by ChemiDoc MP Imaging System (Biorad). Prestained see blue plus ladder was used to estimate proteins´ molecular weight and visualized under normal white light (D).

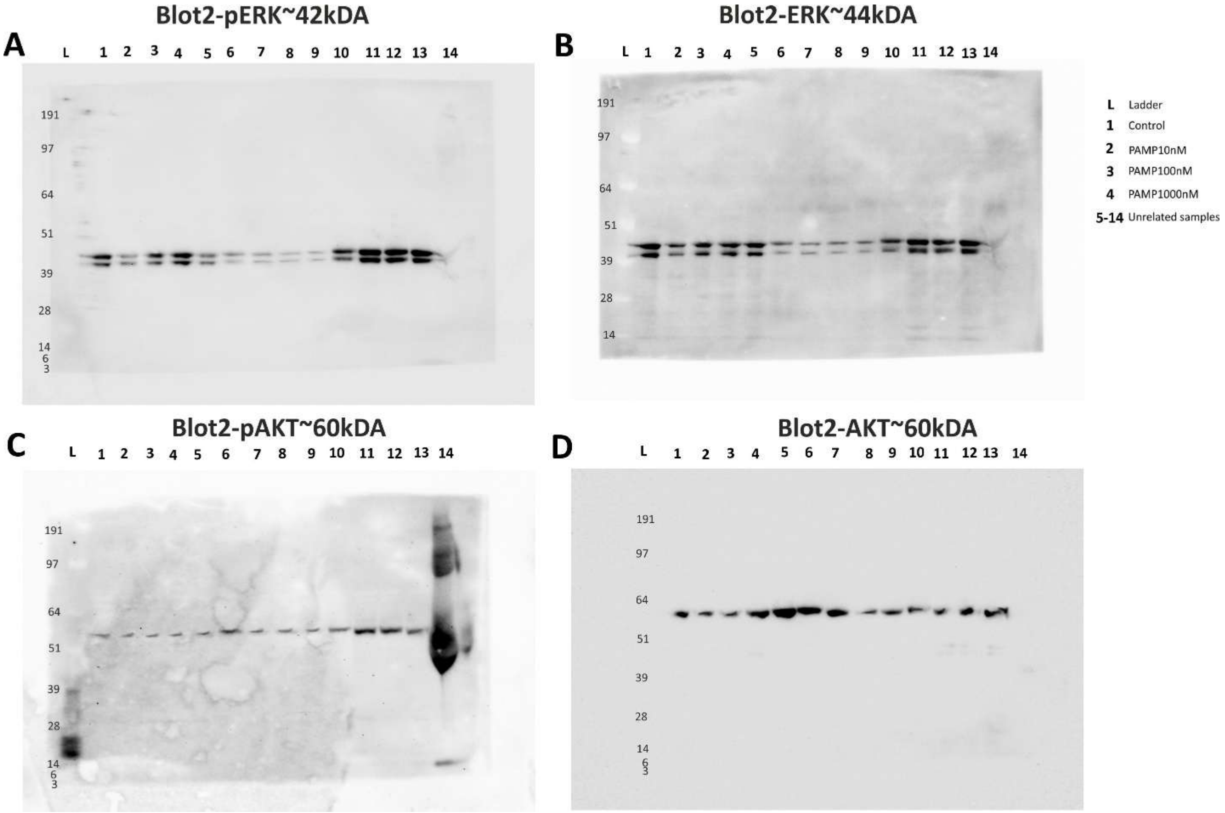

Suppl. Figure S6: Representative Western blot (full) of 5555 cell lysate analysis (A, B, C and D) after 24hrs of incubation with increasing concentrations of PAMP, as indicated. The blots were further incubated with respective antibodies to develop the specific bands and images were taken by ChemiDoc MP Imaging System (Biorad). Prestained see blue plus ladder was used to estimate proteins´ molecular weight and visualized under normal white light (see Fig. S6D).

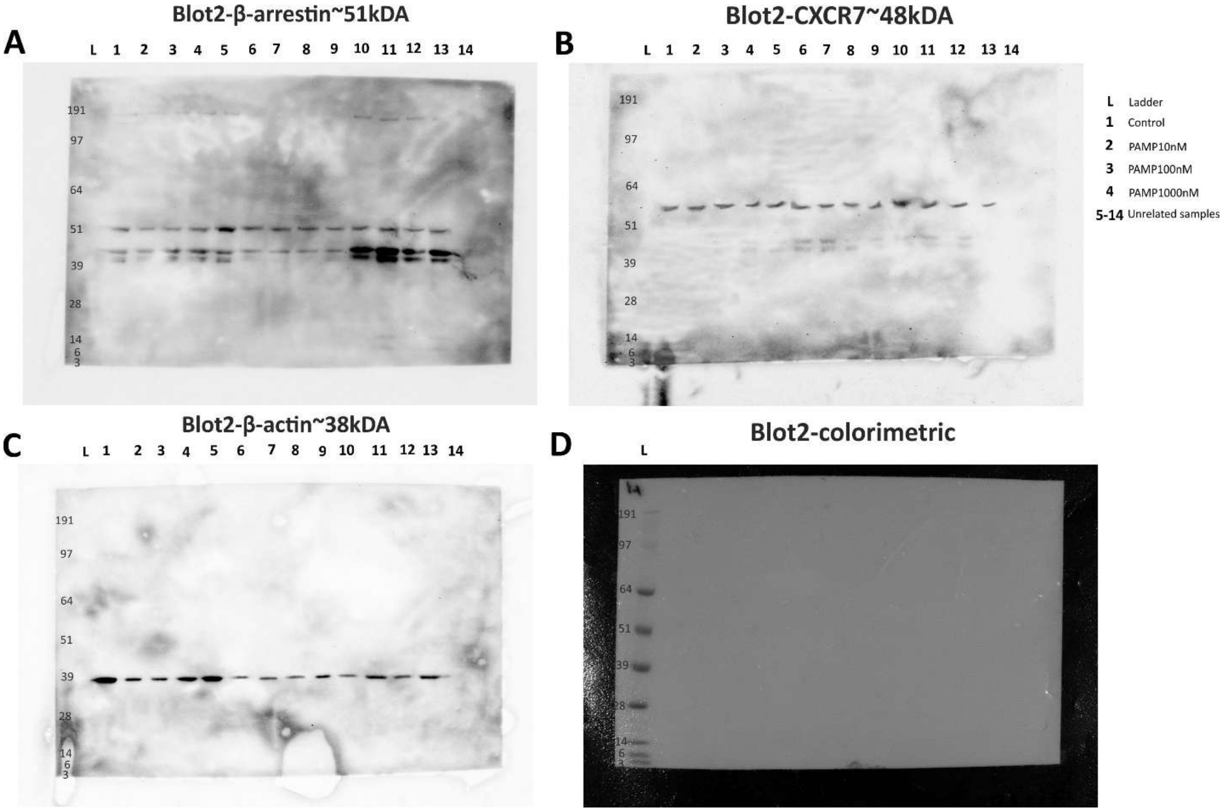

Suppl. Figure S7: Representative Western blot (full) of 5555 cell lysate analysis (A,B,C) after 24hrs of incubation with increasing concentrations of PAMP, as indicated. The blots were further incubated with respective antibodies to develop the specific bands and images were taken by ChemiDoc MP Imaging System (Biorad). Prestained see blue plus ladder was used to estimate proteins´ molecular weight and visualized under normal white light (D).

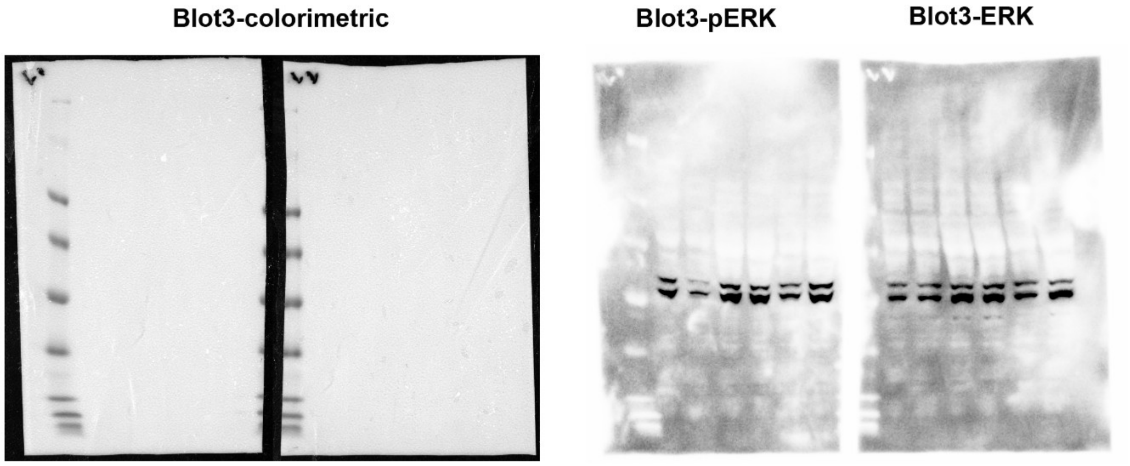

Suppl. Figure S8: Western blot (full) of cell line 5555 exposed to PAMP and receptor inhibitors. Prestained see blue plus ladder (left) was used to estimate proteins´ molecular weight and visualized under normal white light.

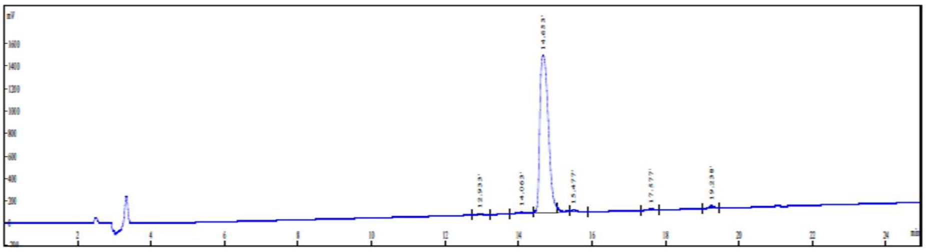

MASS SPECTROMETRY REPORT

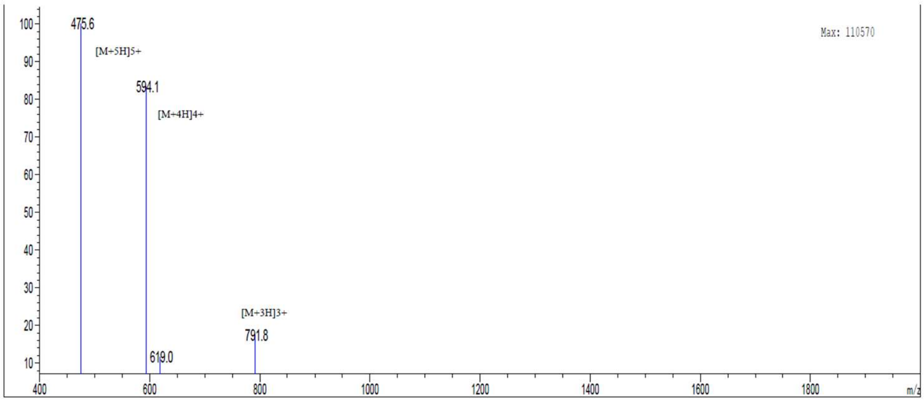

Sample Description		Instrument	Agilent-6125B		
Analyzed date:	2021-11-24	Probe:	ESI	Probe Bias:	+4.5kv
Analyst:	YU	Nebulizer Gas Flow:	1.5L/min	Detector:	1.5kv
Sample:	AR-20-NH2	CDL:	-20.0v	T. Flow:	0.2ml/min
M.W.:	2372.69	CDL Temp.:	250 °C	B. Conc.:	50%H2O/50%ACN
Lot. No.:	P211115-CL949150	Block Temp.:	200 °C		

Suppl. Figure S9: Characterization of the synthetic mouse PAMP peptide. An HPLC profile (above) and a mass spectrometry report (below) were obtained. The mass of the synthetic peptide (2372.69 Da) coincides with the expected mass.

Supplementary tables

PAMP-CXCR7		
**Cluster**	**Size**	**Pose score**
1	155	-894.77
2	146	-873.68
3	101	-894.11
4	82	-897.34
5	67	899.31
6	63	-919.05
7	59	-889.08
8	50	-887.09
9	43	-926.51
10	6	-878.21

Table S1: The score cards of the top 10 clusters for PAMP-CXCR7, docking was evaluated by MASTERO-PIPER based molecular docking.

AM-CXCR7		
**Cluster**	**Size**	**Pose score**
1	95	-974.44
2	95	-1143.32
3	89	-992.50
4	72	-952.22
5	48	-977.03
6	48	-939.15
7	44	-944.62
8	41	-963.13
9	40	-954.10
10	27	-950.89

Table S2: The score cards of the top 10 clusters for AM-CXCR7, docking was evaluated by MASTERO-PIPER based molecular docking.

PAMP-CXCR4		
**Cluster**	**Size**	**Pose score**
1	170	-897.99
2	106	-949.26
3	58	-892.03
4	56	-882.85
5	55	882.75
6	49	-869.37
7	49	-883.09
8	48	-905.17
9	35	-866.88
10	26	-860.62

Table S3: The score cards of the top 10 clusters for PAMP-CXCR4, docking was evaluated by MASTERO-PIPER based molecular docking.

AM-CXCR4		
**Cluster**	**Size**	**Pose score**
1	84	-1028.03
2	75	-881.85
3	56	-869.84
4	54	-922.34
5	50	-867.38
6	48	-1001.15
7	45	-870.93
8	39	-934.79
9	38	-873.26
10	12	-883.24

Table S4: The score cards of the top 10 clusters for AM-CXCR4, docking was evaluated by MASTERO-PIPER based molecular docking.
